# Bone Marrow Mesenchymal Stem Cell‐Derived Exosomes Promote M2 Polarization and Protect Against Acute Lung Injury

**DOI:** 10.1096/fj.202502772R

**Published:** 2025-12-15

**Authors:** Yongmei Cao, Weifeng Huang, Jiawei Shang, Feng Ping, Qin Tan, Wei Wang, Yingchuan Li, Kaixuan Feng

**Affiliations:** ^1^ Department of Critical Care Medicine Shanghai Tenth People's Hospital, Tongji University School of Medicine Shanghai China; ^2^ Department of Critical Care Medcine Shanghai Sixth People's Hospital Affiliated to Shanghai Jiao Tong University School of Medicine Shanghai China; ^3^ Department of Critical Care Medcine Jinshan Branch of Shanghai Sixth People's Hospital Shanghai China

**Keywords:** acute lung injury, alveolar macrophages, BMSCs‐Exos, M2 polarization, miR‐137‐3p

## Abstract

The purpose of this research is to elucidate the molecular mechanisms by which bone marrow mesenchymal stem cell‐derived exosomes (BMSCs‐Exos) improve acute lung injury (ALI) through the regulation of alveolar macrophage polarization. BMSCs‐Exos were prepared and used to pretreat mouse alveolar macrophages (MH‐S), followed by stimulation with LPS and IFN‐γ. The binding interaction between miR‐137‐3p and the 3′ untranslated region (3′UTR) of TRIM24 mRNA was confirmed through luciferase reporter assays. BMSCs‐Exos were used to treat MH‐S cells stimulated with LPS and IFN‐γ, then TRIM24 protein expression, STAT6 acetylation, and reactive oxygen species (ROS) production were analyzed, along with the mRNA levels of macrophage polarization‐related genes. The ALI mouse model was established by intratracheal instillation of LPS, followed by intratracheal administration of BMSCs‐Exos. Subsequently, lung histopathology, pulmonary function, wet‐to‐dry weight ratio and the levels of inflammatory cytokines in bronchoalveolar lavage fluid (BALF) were evaluated. LPS and IFN‐γ stimulation significantly increased the levels of TNF‐α, IL‐1β, and IL‐10 in culture supernatants, as well as CD86 and CD163 mRNA expressions in MH‐S cells. Treatment with BMSCs‐Exos significantly decreased TNF‐α, IL‐1β, and CD86 levels while increasing CD163 and IL‐10 levels. MiR‐137‐3p inhibits TRIM24 protein expression by binding to the 3′UTR of its mRNA. BMSCs upregulated miR‐137‐3p and suppressed TRIM24 protein expression in MH‐S cells co‐cultured with BMSCs, effects that were abolished by GW4869 treatment or by silencing miR‐137‐3p in BMSCs. In LPS and IFN‐y‐induced MH‐S cells, treatment with BMSCs‐Exos effectively upregulated miR‐137‐3p levels and downregulated the TRIM24 expression, which in turn promoted the expression of M2 polarization‐related genes Arg1 and Fizz1, while inhibiting the expression of the M1 polarization gene Nos2 and reducing ROS production. In vivo, intratracheal administration of BMSCs‐Exos alleviated pulmonary inflammation and injury in ALI mice and enhanced M2 polarization of alveolar macrophages. BMSCs‐Exos promote M2 polarization and inhibit M1 polarization of alveolar macrophages by delivering miR‐137‐3p, thereby significantly improving lung injury.

## Graphical abstract

This study elucidates how BMSCs‐Exos mitigate acute lung injury. (1) Exosomal miR‐137‐3p is transferred to alveolar macrophages. (2) It binds TRIM24 mRNA 3'UTR to suppress its expression, promoting STAT6 acetylation. (3) This drives a phenotypic switch from M1 to M2.

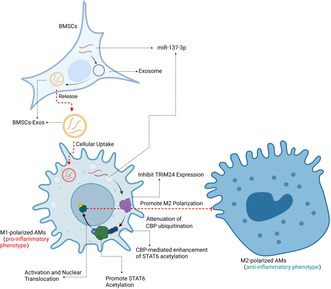

## 1 Introduction

Acute lung injury (ALI) is a common critical illness with an extremely high mortality rate, seriously threatening the lives of critically ill patients and affecting their quality of life [1]. Pulmonary macrophages, including alveolar macrophages, interstitial macrophages, and bronchial macrophages, act as immune sentinels that maintain lung homeostasis. Alveolar macrophages (AM), which account for over 90% of pulmonary macrophages, play a central role in regulating inflammation and immune imbalance in ALI [2]. During ALI, AM undergo phenotypic transformation into either pro‐inflammatory M1 or anti‐inflammatory M2 macrophages, depending on the lung microenvironment [3]. M1 macrophages produce pro‐inflammatory mediators to exert a defensive effect, while M2 macrophages release anti‐inflammatory factors and promote tissue repair. Studies have shown that M2 polarization of AMs can attenuate lung injury and fibrosis by suppressing the inflammatory cascade and cytokine storm [4].

Although promoting the M2 polarization of AM shows great promise for anti‐inflammatory and tissue repair therapies in ALI, its clinical translation remains hindered by several challenges. The complex pulmonary microenvironment compromises the efficacy and specificity of conventional approaches—such as cytokine stimulation, chemical agents, and stem cell therapy, which resulted in inconsistent M2 induction and limited regulatory ability on macrophage phenotype transformation [5]. Moreover, the high plasticity and reversibility of AM phenotypes make it difficult to achieve sustained therapeutic effects through single‐pathway modulation. Excessive M2 polarization may also cause immunosuppression, thereby increasing the risk of secondary infections and tumor development, further limiting its clinical application [6]. Considering the rapid progression and narrow therapeutic window of ALI, the development of precise and targeted strategies to effectively induce M2 polarization in AM is both critical and urgent for reshaping the immune microenvironment and improving clinical outcomes in ALI [7].

Mesenchymal stem cells (MSCs), exhibit strong self‐renewal and multi‐lineage differentiation abilities, making them a key candidate for ALI biological therapies [8]. Human bone marrow‐derived MSCs (BMSCs), derived from adult bone marrow, have demonstrated protective effects against LPS‐induced lung injury in mice [9]. Studies have shown that BMSCs were taken up by the alveolar macrophages and exerted immunoregulatory effects and thereby improved survival rates [10]. BMSCs could restore the balance of M1/M2 macrophages, thereby reducing lung injury, primarily through modulating immune cells, and exerting paracrine/autocrine effects [11]. Some studies emphasized the direct immunomodulatory role of BMSCs, while others highlighted that secreted factors are the main therapeutic mechanism. Additionally, MSC‐derived exosomes (MSCs‐Exos) have been shown to influence macrophage polarization by delivering miRNAs [12, 13]. In this study, we report for the first time that bone marrow mesenchymal stem cell‐derived exosomes (BMSCs‐Exos) deliver miR‐137‐3p to AM, suppressing M1 polarization and promoting M2 polarization by downregulating TRIM24 and enhancing STAT6 acetylation. These findings provide new insights into the BMSCs‐mediated regulation of lung inflammation in ALI and offer theoretical support for BMSCs‐based therapies.

## 2 Methods

### 2.1 Preparation of BMSCs‐Derived Exosomes (BMSCs‐Exos)

BMSCs were isolated from 6‐week‐old male C57BL/6 mice (Shanghai Slake Experimental Animal Co. Ltd., Shanghai, China) as previously described [14]. Briefly, bone marrow was flushed from tibias and femurs using Minimum Essential Medium (MEM; 11095080, Gibco, Thermo Scientific, CA, USA) with 10% exosome‐free fetal Bovine Serum (FBS; Exoperfect, Shanghai, China), 100 U/mL penicillin, and 100 μg/mL streptomycin. The suspension was centrifuged at 2000× *g* for 2 min, and red blood cells were lysed. After a second centrifugation, cells were resuspended in the aforementioned complete MEM, and cultured at 37°C with 5% CO₂. Medium was changed every 3 days. On day 9, adherent cells were trypsinized and passaged at 75% confluency. The second‐passage BMSCs were morphologically examined under a fluorescence inverted microscope and characterized by flow cytometry as positive for CD105, CD90, and CD73, but negative for CD34. For flow cytometric analysis, cells were fixed with 1% methanol, blocked with 1% BSA and 0.1% goat serum, incubated with antibodies anti‐mouse CD105, CD90, CD73 and CD34 (ab314950/ab25322/FAB4488V/ab23830; Abcam, Cambridge, UK or R&D Systems, MN, USA) each diluted 1:250, then washed and resuspended in Dulbecco's Phosphate‐Buffered Saline (dPBS). BMSCs‐Exos were isolated as previously described [15]. Briefly, fourth‐passage BMSCs were cultured for 48 h, and the supernatant was collected and centrifuged at 500 × g (10 min), 12 000× *g* (20 min), and 100 000× *g* at 4°C (120 min). The final pellet was resuspended in 200 μL of dPBS and stored at −80°C until further use. The protein concentration in 1 μL of exosomes was quantified using a bicinchoninic acid (BCA) assay kit according to the manufacturer's instructions (23225, Pierce, Thermo Scientific). The morphology of the extracted exosomes was observed using transmission electron microscopy (H‐600 HITACHI, Japan) and nanoparticle tracking analysis (NTA). For TEM, exosomes were fixed with 2% paraformaldehyde, applied to carbon‐coated grids, stained with 2% uranyl acetate, and imaged with a 120 kV TEM [16]. Exosomal proteins were extracted using the Total Exosome RNA and Protein Isolation Kit (4478545, Invitrogen, Thermo Scientific) and analyzed for CD9, CD63, and CD81 protein expression by western blotting. Rabbit anti‐mouse CD9, CD63, and CD81 primary antibodies (ab223052/ab216130, Abcam; 10 037, CST, MA, USA) were diluted at 1:400, 1:400, and 1:600, respectively. The HRP‐conjugated goat anti‐rabbit secondary antibody (ab205718, Abcam) was diluted at 1:3000.

### 2.2 BMSCs‐Exos and LPS + IFN‐γ Treatment of Mouse Alveolar Macrophages

The mouse alveolar macrophage cell line (MH‐S; ABI‐TC4178, NJ, USA) was cultured in complete MEM at 37°C and 5% CO_2_, with the cells growing adherently. When the cell density reached 90%, they were digested with 0.25% trypsin containing EDTA and seeded into 6‐well plates at a density of 5 × 10^4^ cells per well. After overnight incubation to allow for complete cell adhesion, the cells were divided into four groups: the MH‐S group, which was not treated; the LPS + IFN‐γ group, where LPS and IFN‐γ were used to induce M1 polarization in MH‐S cells; and the BMSCs‐Exos + LPS + IFN‐γ group, where BMSCs‐Exos at concentrations of 10, 30, and 100 μg/mL were used for pretreatment followed by LPS and IFN‐γ induction. LPS and IFN‐γ (L2880/IF005, Sigma‐Aldrich, MO, USA) were applied at final concentrations of 100 and 20 ng/mL, respectively, for a subsequent 24 h period. BMSCs‐Exos were added to the culture medium 1 h before LPS and IFN‐γ induction. After completing the treatments, the cells were collected, total RNA was extracted, and the mRNA levels of CD86 and CD163 were analyzed by Reverse Transcription Quantitative Polymerase Chain Reaction (RT‐qPCR). The concentrations of TNF‐α, IL‐1β, and IL‐10 in the cell supernatants were quantified using ELISA kits (BMS607‐3, BMS6002‐2, and BMS614, Thermo Scientific) according to the manufacturer's protocols.

### 2.3 BMSCs‐Exos Treatment or Co‐Culture With BMSCs of LPS + IFN‐γ Induced MH‐S Cells

The experiment was divided into 7 groups: (1) MH‐S group, where MH‐S cells were cultured individually without treatment; (2) MH‐S + LPS + IFN‐γ group, where MH‐S cells were cultured individually and induced with LPS and IFN‐γ; (3) BMSCs/MH‐S + LPS + IFN‐γ group, BMSCs were co‐cultured with MH‐S cells, and LPS and IFN‐γ were added to the upper chamber where the MH‐S cells were located; (4)BMSCs+siRNA‐Pri‐miR‐137/MH‐S + LPS + IFN‐γ group, BMSCs transfected with siRNA‐Pri‐miR‐137 were co‐cultured with MH‐S cells, and LPS and IFN‐γ were added to the upper chamber where the MH‐S cells were located; (5) BMSCs + GW4869/MH‐S + LPS + IFN‐γ group: BMSCs were co‐cultured with MH‐S cells, and LPS and IFN‐γ were added to the upper chamber containing the MH‐S cells, while GW4869 was added to the lower chamber; (6) BMSCs‐Exos (10 μg/mL) + MH‐S + LPS + IFN‐γ group, MH‐S cells were cultured alone, and BMSCs‐Exos were added to the culture medium at a final concentration of 10 μg/mL; (7) BMSCs‐Exos (100 μg/mL) + MH‐S + LPS + IFN‐γ group, MH‐S cells were cultured alone, and BMSCs‐Exos were added to the culture medium at a final concentration of 100 μg/mL. The co‐culture of BMSCs and MH‐S cells was conducted using a Trans‐well system. The third generation of BMSCs and MH‐S cells was seeded into the lower and upper chambers at densities of 1 × 10^5^ and 5 × 10^4^ cells, respectively. The GW4869 (D1692, Sigma‐Aldrich), a potent inhibitor of exosome biogenesis and secretion, was added to the lower chamber at a final concentration of 20 μM at the same time. A siRNA (5′‐GUAAUCCGUAUUAUCCACCtt‐3′) targeting mouse pri‐miR‐137 was synthesized and transiently transfected into BMSCs using Lipofectamine 3000 (Invitrogen, Thermo Scientific) according to the manufacturer's instructions. After 24 h of transfection, BMSCs were trypsinized, reseeded, and co‐cultured with MH‐S cells. After incubation for 48 h, the MH‐S cells in the upper chamber were harvested for total RNA extraction to measure TRIM24 mRNA and miR‐137‐3p levels using RT‐qPCR. Total protein was also extracted to determine TRIM24 protein expression via western blotting. In addition, the MH‐S cells in the upper chamber were used for miR‐137‐3p detection by fluorescence in situ hybridization.

### 2.4 Fluorescence In Situ Hybridization (FISH) Assay

FISH assay was performed to detect the intracellular localization and expression of miR‐137‐3p in MH‐S cells. After appropriate treatment, cells were fixed with 4% paraformaldehyde for 15 min at room temperature, followed by permeabilization with 0.1% Triton X‐100 for 10 min. After rinsing with dPBS, a Cy3‐labeled and LNA‐modified miR‐137‐3p‐specific probe (5′‐Cy3‐CTACGCGTATTCTTAAGCAATAA‐3′) was hybridized to the cells in pre‐warmed hybridization buffer (containing formamide) at 37°C for 15 h in a humidified, light‐protected chamber. After hybridization, cells were sequentially washed with 2 × SSC, 1 × SSC, and 0.5 × SSC buffers at 37°C to remove unbound probe. Nuclei were counterstained with DAPI for 5 min, and slides were mounted with anti‐fade mounting medium. Fluorescent signals were visualized using a fluorescence or confocal microscope. The red fluorescence (Cy3) indicated miR‐137‐3p expression, and DAPI‐stained nuclei served as references. Image acquisition and quantification were performed using ImageJ or equivalent image analysis software. Negative control slides hybridized with a scrambled probe were included to confirm probe specificity. All procedures were carried out under RNase‐free conditions.

### 2.5 Luciferase Assay

TargetScan (version 7.1) was utilized to predict the binding sites of miR‐137‐3p on the 3′ untranslated region (UTR) of mouse TRIM24 mRNA. Following this, luciferase reporter vectors, pGL3‐TRIM24‐3′UTR (wt), which contain the wild‐type binding site for miR‐137‐3p (5′‐AGCAAUA‐3′), and pGL3‐TRIM24‐3′UTR (mt), which contains a mutated binding site (5′‐CAAUAGA‐3′), were constructed. These vectors were co‐transfected into 293 cells that were seeded in a 24‐well plate at a density of 1 × 10^4^ cells per well, along with miR‐137‐3p mimics (5′‐GAUGCGCAUAAGAAUUCGUUAUUtt‐3′), miR‐137‐3p inhibitors, and miR‐137‐3p scramble, respectively. After 48 h of incubation, luciferase activity was measured using the Dual‐Luciferase Reporter Assay System (E1910, Promega, USA) and normalized to renilla luciferase levels.

### 2.6 The TRIM24 Recovery Experiment

The experimental groups were as follows: the Control group (no treatment); the BMSCs‐Exos group (MH‐S cells co‐cultured with 10, 30, and 100 μg/mL BMSCs‐Exosomes for 48 h); and the Lv‐TRIM24+ BMSCs‐Exos (100 μg/mL) group (MH‐S cells infected with recombinant lentivirus Lv‐TRIM24, then co‐cultured with 100 μg/mL BMSCs‐Exosomes for 48 h). The recombinant lentivirus Lv‐TRIM24 was administered 24 h before BMSCs‐Exos treatment at a multiplicity of infection (MOI) of 5. Lv‐TRIM24, which contains a CMV promoter and the coding region of the mouse TRIM24 gene, was used to overexpress exogenous TRIM24 in MH‐S cells. By utilizing a universal promoter instead of the TRIM24 promoter, its expression is not regulated by miR‐137‐3p, making it suitable as a recovery control for pathway validation. After treatment, MH‐S cells were collected and analyzed for the expression of TRIM24 and phosphorylated STAT6 proteins by Western blotting. Rabbit anti‐mouse TRIM24, phosphorylated STAT6, and β‐actin primary antibodies (ab211300/ab263947/ab179467, Abcam) were diluted at 1:500, 1:600, 1:400, and 1:2000, respectively. The HRP‐conjugated goat anti‐rabbit secondary antibody was diluted at 1:3000. Additionally, these MH‐S cells were used to measure Reactive Oxygen Species (ROS) levels and the mRNA expression of macrophage polarization markers Nos2, Arg1, and Fizz1, detected by fluorescence probe assay and RT‐qPCR, respectively.

### 2.7 Reactive Oxygen Species Assay

After treatment, MH‐S cells were incubated with a DCFH‐DA probe, which is oxidized by ROS to form highly fluorescent DCF. The cells were then washed to remove excess probe, and fluorescence intensity, which correlates with intracellular ROS levels, was measured using a fluorescence microplate reader with an excitation wavelength of 485 nm and an emission wavelength of 530 nm. ROS activity was quantified by comparing fluorescence intensity across the experimental groups.

### 2.8 STAT6 Protein Acetylation Assay

To assess the effect of BMSCs‐Exos delivering miR‐137‐3p on STAT6 activation by inhibiting TRIM24, MH‐S cells were treated with BMSCs‐Exos at a final concentration of 100 μg/mL for 48 h. For immunoprecipitation, MH‐S cells were first transfected with a STAT6 expression vector, pcDNA‐HA‐STAT6, which carries an HA tag (YPYDVPDYA). After BMSCs‐Exos treatment, the cells were harvested and lysed using Pierce IP Lysis Buffer (87787, Thermo Scientific). The lysates were then subjected to immunoprecipitation using 4 μg of anti‐HA antibody or normal rabbit IgG (ab9100/ab125938, Abcam), together with protein A‐Sepharose (Santa Cruz Biotechnology, Texas, USA). The immune complexes were washed and analyzed by immunoblotting with anti‐STAT6 (1:800) and anti‐pan Acetylation (66289, Proteintech, USA) (1:500) antibodies.

### 2.9 Animal Experiments

All animal procedures were approved by the Ethics Committee of Shanghai Tenth People's Hospital, Tongji University School of Medicine (Approval No. SHDSYY‐2023‐6632), and conducted in accordance with the guidelines for the ethical treatment of laboratory animals. All efforts were made to minimize animal suffering and reduce the number of animals used. Specific pathogen‐free (SPF) grade male C57BL/6 mice (6 weeks old, weighing 18–20 g) were housed under controlled environmental conditions (22°C ± 2°C, 12 h light/dark cycle, 50%–60% humidity) with free access to food and water for one week prior to experimentation for acclimatization. ALI was induced via intratracheal instillation of LPS. Briefly, mice were anesthetized, placed in a supine position, and the trachea was surgically exposed. LPS (5 mg/kg body weight, dissolved in 50 μL of sterile dPBS) was instilled into the trachea to induce ALI. The peak inflammatory response was observed 24 h post‐instillation. To assess the therapeutic efficacy of exosomes, BMSC‐Exos were injected via the tail vein 2 h after LPS exposure at doses of 1 or 10 mg/kg body weight (total protein basis) in 50 μL of sterile dPBS. Mice were euthanized 24 h after exosome administration for sample collection and analysis. Lung tissues were harvested, fixed in formalin, embedded in paraffin, sectioned, and stained with hematoxylin and eosin (H&E) to evaluate histopathological changes under a microscope. To ensure accuracy, five random fields from each group were assessed. The main evaluation criteria include neutrophil infiltration (in the alveolar or the interstitial space), hyaline membranes, proteinaceous debris filling the airspaces, and septal thickening. Lung injury was scored based on established criteria [17, 18], the specific scoring criteria are shown in Table 1. The formula for calculating the final score is as follows: The final score = [(20 × A) + (14 × B) + (7 × C) + (7 × D) + (2 × E)]/(number of fields×100). Pulmonary function was assessed using a small‐animal ventilator system (flexiVent, SCIREQ, Canada), with forced expiratory volume in 0.1 s to forced vital capacity (FEV₀.₁/FVC%) as a primary readout. Lung edema was evaluated by calculating the wet‐to‐dry (W/D) weight ratio: the left lung was weighed immediately after removal (wet weight), then dried at 60°C for 48 h before weighing again (dry weight). Bronchoalveolar lavage fluid (BALF) was collected to assess inflammatory cytokine levels. Concentrations of TNF‐α, IL‐1β, and IL‐10 were quantified using ELISA kits following the manufacturer's protocols. Following repeated model induction and exosome treatment, lungs were harvested and alveolar macrophages were isolated. Total RNA was extracted, and RT‐qPCR was performed to quantify Nos2 and Arg1 mRNA, canonical markers of M1 and M2 polarization, respectively, to evaluate the polarization status of alveolar macrophages across experimental groups.

**TABLE 1:** Lung injury scoring criteria.

Parameter	Score per field		
	0	1	2
A. Neutrophils in the alveolar space	None	1–5	> 5
B. Neutrophils in the interstitial space	None	1–5	> 5
C. Hyaline membranes	None	1	> 1
D. Proteinaceous debris filling the airspaces	None	1	> 1
E. Alveolar septal thickness (× baseline)	< 2	2–4	> 4

### 2.10 RT‐qPCR

Total RNA was extracted using Trizol (15596026.Thermo Scientific). One microgram of RNA was reverse‐transcribed into cDNA with a reverse transcription kit, using random primers for U6 and stem‐loop primers (5′‐GTCGTATCCAGTGCAGGGTCCGAGGTATTCGCACTGGATACGACAATAAC) for miR‐137‐3p. RT‐qPCR was performed using SYBR Green Mix (A57155, Applied Biosystems, CA, USA) on the Applied Biosystems 7500 Fast System, with 2 μL cDNA template in a 20 μL reaction (10 μL SYBR premix, 0.2 μL each primer (20 μM), 7.6 μL dH_2_O). PCR conditions: 95°C/10 s, 60°C/10 s, 72°C/20 s, 40 cycles. MiR‐137‐3p expression was calculated using 2^−ΔCT^. For mRNA quantification, cDNA was synthesized using Oligo(dT) and Random 9‐mer primers (Takara, Dalian, China), with β‐actin as the internal reference. All primer sequences used for PCR are shown in Table 2.

**TABLE 2:** Primer information for PCR.

Target	Primer seqence	
miR‐137‐3p	F	5′‐GTCGTTATCCAGGTGCGTGTCGT‐3′
	R	5′‐GATGCGCATAAGAATTCGTTATT‐3′
U6	F	5′‐GTGCTCGCTTCGGCAGCACATA‐3′
	R	5′‐ATATGGAACGCTTCACGAA‐3
TRIM24	F	5′‐CCGGTGGGAGGGTCTTACAAT‐3′
	R	5′‐CCTCGGCTGGGCGTGAT‐3′
Nos2	F	5′‐TTTGCCACGGACGAGACGGATAGG‐3′
	R	5′‐CGGGCACATGCAAGGAAGGGAACT‐3′
Arg1	F	5′‐GGATTGGCAAGGTGATGGAAGAGA‐3′
	R	5′‐GGTCGCCGGGGTGAATG‐3′
Fizz1	F	5′‐CAATCCAGCTAACTATCCCTCCAC‐3
	R	5′‐AAGCCACAAGCACACCCAGTAGC‐3
CD163	F	5′‐CTGGCGGGTGGTGAAAACAACT‐3′
	R	5′‐GGCAAACCACGGACACTTCATTC‐3′
CD86	F	5′‐GTCACCCGAAACCTAAGAA‐3′
	R	5′‐ATCCGGGAATGAAAGAGA‐3′
β‐actin	F	5′‐GACGATGCTCCCCGGGCTGTATTC‐3′
	R	5′‐TCTCTTGCTCTGGGCCTCGTCACC‐3′

### 2.11 Western Blot

Total proteins were extracted from cells and exosomes using RIPA lysis buffer (Thermo Fisher) according to the manufacturer's protocol. Protein samples were separated by 11%–12% SDS‐PAGE, and target bands were identified based on molecular weight. After electrophoresis, proteins were transferred to PVDF membranes (Millipore, MA, USA) using a multi‐functional semi‐dry transfer membrane instrument. Target protein regions were determined using pre‐stained molecular weight markers, and the membrane was trimmed accordingly. The membranes were blocked with 5% non‐fat milk in TBST buffer for 1 h at room temperature and then probed overnight at 4°C with primary antibodies. After washing, membranes were incubated with HRP‐conjugated secondary antibody at room temperature for 1 h. Protein bands were detected using an ECL substrate (34579, Pierce, Thermo Scientific) and exposed to X‐ray film. The developed films were scanned, and band intensities were quantified using ImageJ software.

### 2.12 Statistical Analyses

The normality distribution of all data was tested using the Shapiro–Wilk test and statistically analyzed with SPSS 18.0 software. Results are expressed as mean ± standard deviation (SD). Inter‐group comparisons were performed using Student's *t*‐test for two groups or one‐way ANOVA for multiple groups. The statistical significance was set at *p* < 0.05, indicated by an asterisk (*).

## 3 Results

### 3.1 Characterization of BMSCs‐Exos

**FIGURE 1:**
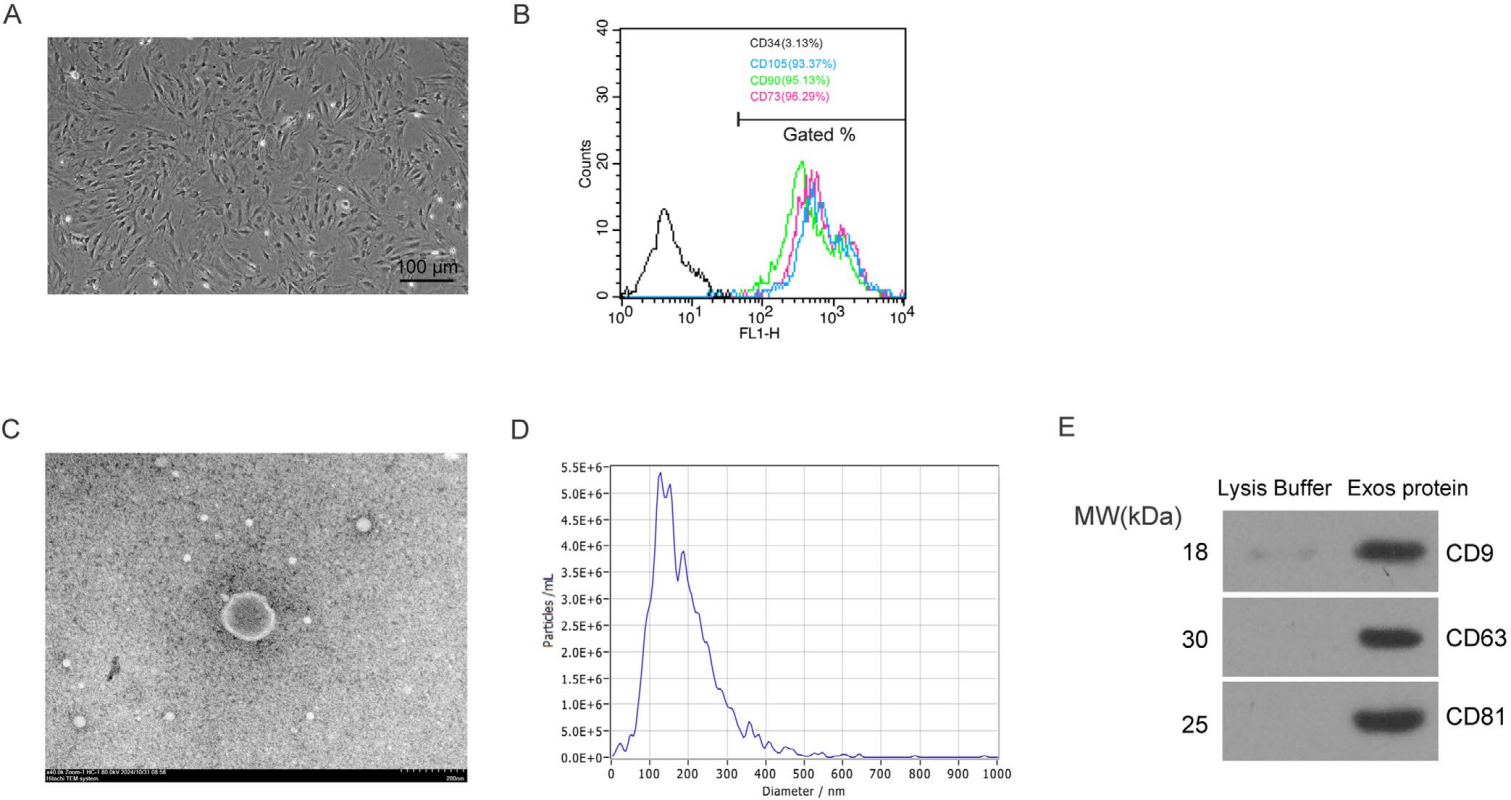
Preparation of mouse BMSCs and their exosomes. (A) Morphological observation of mouse BMSCs at 120× magnification. To ensure objectivity, five random fields were selected for observation in each analysis. (B) Flow cytometry analysis of the expression of surface markers CD105, CD90, CD73 and CD34 in passage 2 BMSCs. (C) SEM characterization of BMSCs‐Exos. (D) Particle size analysis of BMSCs‐Exos using NTA. (E) Western blot analysis of CD9, CD63, and CD81 protein expression in BMSCs‐Exos, with lysis buffer used as a negative control. Three biological replicates were performed for the experiment (*n* = 3), and each replicate was tested three times.

At passage 2, BMSCs exhibited adherent growth in a monolayer with a predominantly spindle‐shaped morphology, and the cell diameter ranged from 35 to 70 μm (Figure 1A). Flow cytometric analysis confirmed that over 90% of the cells were positive for the mesenchymal surface markers CD105 CD90 and CD73, while fewer than 4% of the cells expressed the hematopoietic marker CD34 (Figure 1B). TEM revealed that the isolated exosomes displayed the characteristic cup‐shaped morphology, with diameters ranging from 50 to 100 nm (Figure 1C). NTA showed that the extracellular vesicles had an average diameter of approximately 150 nm, with the majority of particles distributed between 130 and 180 nm (Figure 1D). Western blot analysis further confirmed the presence of exosomal surface markers CD9, CD63, and CD81 in the protein extracts derived from BMSC‐exos (Figure 1E).

### 3.2 BMSCs‐Exos Promote M2 and Inhibit M1 Polarization in MH‐S Cells

**FIGURE 2:**
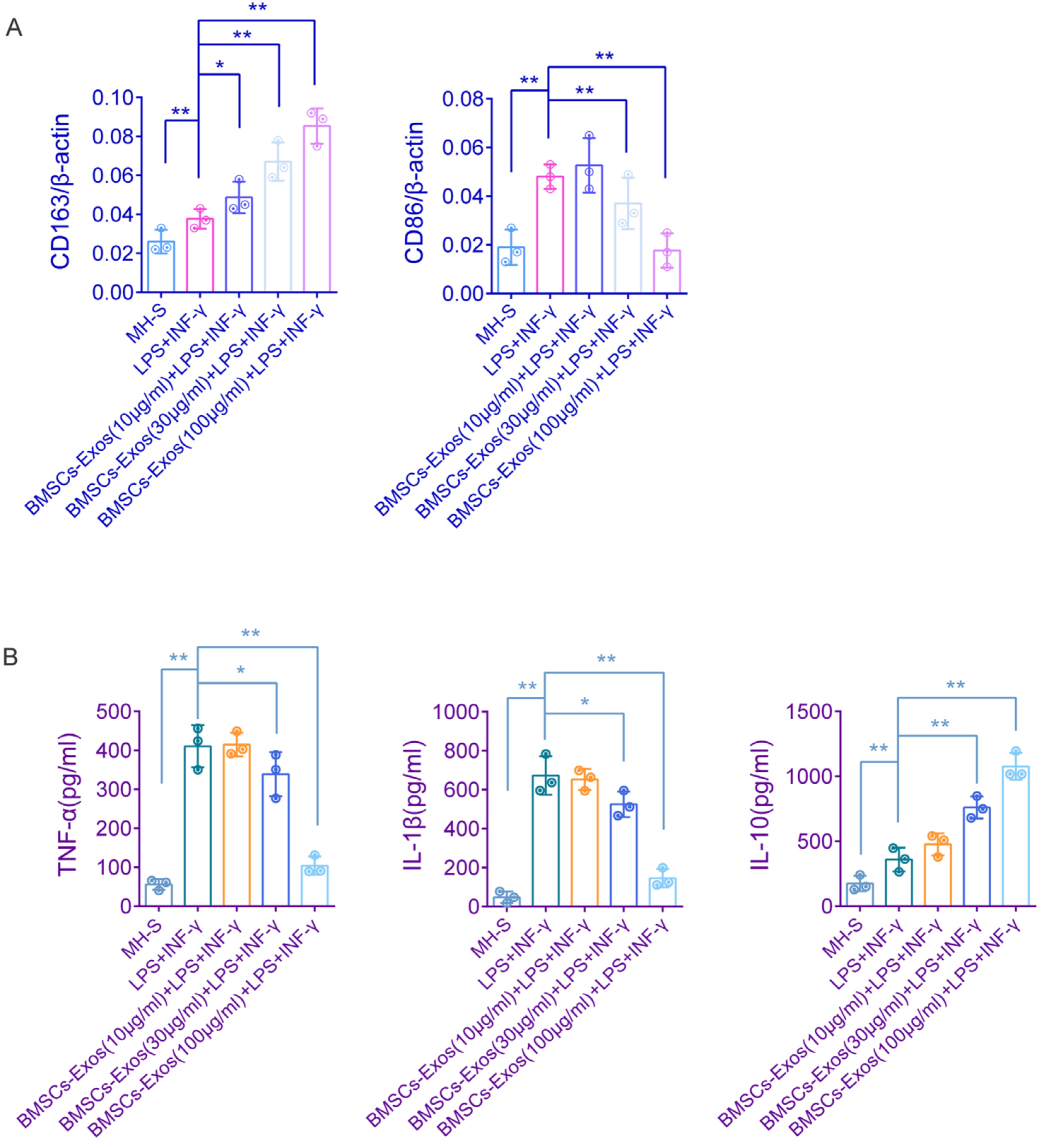
Measurement of macrophage polarization markers and inflammatory factor secretion. (A) RT‐qPCR was used to measure the expression of CD86 and CD163 in MH‐S cells across different treatment groups, with β‐actin as the reference gene. (B) ELISA was performed to assess the levels of inflammatory factors (TNF‐α, IL‐1β, and IL‐10) in the supernatants of MH‐S cells from various treatment groups. All experiments were conducted with three biological replicates (*n* = 3), with two technical replicates per sample, and data are presented as mean ± SD. *p < 0.05, **p < 0.01, versus the specified group. *t*‐test.

RT‐qPCR analysis demonstrated that treatment with LPS and IFN‐γ for 48 h markedly elevated the expression of CD86 (*p* < 0.01 vs. MH‐S group); this increase was significantly suppressed by subsequent treatment with BMSCs‐Exos (*p* < 0.01 for both 30 and 100 μg/mL concentrations vs. LPS + IFN‐γ group). LPS and IFN‐γ treatment significantly enhanced the expression level of CD163 (*p* < 0.01 vs. MH‐S group), which was further augmented by BMSCs‐Exos treatment (*p* < 0.05 at 10 μg/mL and *p* < 0.01 at both 30 and 100 μg/mL concentrations vs. LPS+ IFN‐γ group) (Figure 2A). ELISA results showed that the levels of TNF‐α, IL‐1β, and IL‐10 in MH‐S cell supernatants were significantly increased following LPS and IFN‐γ stimulation (*p* < 0.01 vs. MH‐S group). Subsequent administration of BMSCs‐Exos effectively reduced TNF‐α and IL‐1β levels (*p* < 0.05 at 30 μg/mL and *p* < 0.01 at 100 μg/mL vs. LPS+ IFN‐γ group) and further elevated IL‐10 levels (*p* < 0.01 for both 30 and 100 μg/mL vs. LPS + IFN‐γ group) (Figure 2B). The above results demonstrate that LPS and IFN‐γ induce M1 polarization of macrophages, whereas BMSCs‐Exos attenuate this effect and promote M2 polarization.

### 3.3 Co‐Culture With BMSCs Suppresses TRIM24 Protein Expression in MH‐S Cells via BMSC‐Exos Mediated miR‐137‐3p Transfer

**FIGURE 3:**
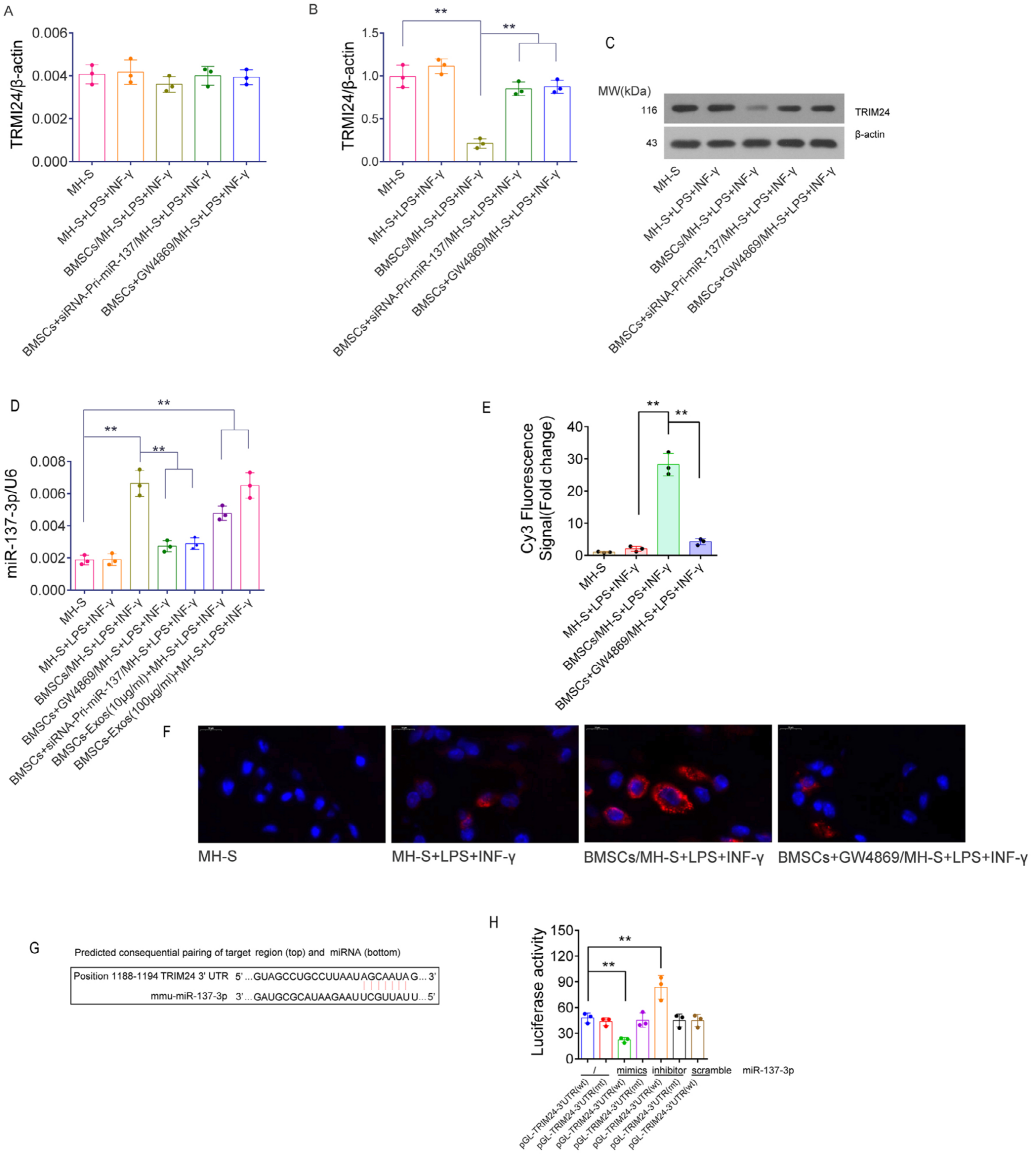
Analysis of miR‐137‐3p expression and TRIM24 expression in MH‐S cells under various treatment conditions, and validation of their regulatory relationship via luciferase assay. (A) RT‐qPCR was used to measure the relative expression of TRIM24 mRNA in MH‐S cells, with β‐actin as the reference gene. (B, C) Western blotting was performed to analyze TRIM24 expression in MH‐S cells across different treatment groups, using β‐actin as an internal control. (D) RT‐qPCR was conducted to measure miR‐137‐3p levels in MH‐S cells, using U6 as a reference. (E, F) FISH analysis was used to detect miR‐137‐3p expression in MH‐S cells from different treatment groups. The red signal represents Cy3‐labeled miR‐137‐3p‐specific binding fluorescent probes. (G) Bioinformatics prediction of the binding site between miR‐137‐3p and the 3′UTR of TRIM24 mRNA. (H) Luciferase assay: 293 cells transfected for 48 h were used to assess luciferase activity, with relative luciferase activity expressed as the ratio of firefly to Renilla luciferase activity. The cells were observed at 200× magnification. All experiments were performed with three biological replicates (*n* = 3), with two technical replicates per sample, and data are presented as mean ± SD. **p < 0.01, versus the specified group. *t*‐test.

RT‐qPCR analysis revealed no significant differences in TRIM24 mRNA expression among the treatment groups (*p* > 0.05). Western blotting results showed that TRIM24 protein expression in the MH‐S + LPS + IFN‐γ group did not differ significantly from that in the MH‐S group (*p* > 0.05). However, compared with the MH‐S + LPS + IFN‐γ group, TRIM24 protein expression was markedly decreased in MH‐S cells of the BMSCs/MH‐S + LPS + IFN‐γ group (*p* < 0.01). Moreover, pretreatment with GW4869 (20 μM) or transfection with siRNA‐Pri‐miR‐137 effectively reversed the BMSC‐mediated reduction in TRIM24 protein expression in MH‐S cells (*p* < 0.01 vs. BMSCs/MH‐S + LPS + IFN‐γ group) (Figure 3A–C).

### 3.4 Mouse BMSCs Express miR‐137‐3p and Transfer It to Co‐Cultured MH‐S Cells Through Exosomes

RT‐qPCR results demonstrated that a 48 h induction with LPS + IFN‐γ had no significant effect on miR‐137‐3p expression in MH‐S cells (*p* > 0.05 vs. the MH‐S group). However, co‐culture with BMSCs markedly upregulated miR‐137‐3p levels in MH‐S cells treated with LPS + IFN‐γ (*p* < 0.01 vs. the MH‐S + LPS + IFN‐γ group). This upregulation was significantly reversed by the addition of GW4869 (20 μM) or transfection of siRNA‐Pri‐miR‐137 in BMSCs, which reduced miR‐137‐3p expression in MH‐S (*p* < 0.01 vs. the BMSCs/MH‐S + LPS+ IFN‐γ group). Furthermore, the addition of BMSC‐Exos can induce a dose‐dependent upregulation of miR‐137‐3p levels in MH‐S cells within the 10–100 μg/mL dosage range (Figure 3D). FISH analysis showed that the red fluorescence signal (emitted by the Cy3‐labeled miR‐137‐3p probe) in MH‐S cells did not differ significantly between the MH‐S group and the MH‐S + LPS + IFN‐γ group (*p* > 0.05). In contrast, co‐culture with BMSCs led to a notable increase in red fluorescence intensity in MH‐S cells exposed to LPS + IFN‐γ (*p* < 0.01 vs. the MH‐S + LPS + IFN‐γ group). This fluorescence enhancement was significantly attenuated upon treatment with GW4869 (20 μM), as evidenced by reduced signal intensity in the BMSCs + GW4869/MH‐S + LPS + IFN‐γ group (*p* < 0.01 vs. the BMSCs/MH‐S + LPS + IFN‐γ group) (Figure 3E,F). These findings collectively indicate that BMSCs serve as a source of miR‐137‐3p and can transfer it to co‐cultured MH‐S cells via exosomes, thereby suppressing TRIM24 protein expression.

### 3.5 MiR‐137‐3p Targets the 3′UTR of TRIM24 mRNA to Inhibit Protein Expression

Bioinformatics predictions identified a 7‐base binding site (5′‐AGCAAUA‐3′) for the miR‐137‐3p seed region in the 3′UTR of mouse TRIM24 mRNA (Figure 3G). The luciferase assay demonstrated that transfection with miR‐137‐3p mimics significantly reduced intracellular luciferase activity compared with the pGL3‐TRIM24‐3′UTR (wt) group (*p* < 0.01). In contrast, miR‐137‐3p inhibitors markedly increased luciferase activity (*p* < 0.01), whereas the miR‐137‐3p scramble had no significant effect in 293 cells transfected with pGL3‐TRIM24‐3′UTR (wt) (*p* > 0.05). However, in cells transfected with the pGL3‐TRIM24‐3′UTR (mt) construct, neither the miR‐137‐3p mimics nor the inhibitors produced any significant change in luciferase activity (*p* > 0.05) (Figure 3H). These results collectively indicate that TRIM24 is a target gene regulated by miR‐137‐3p.

### 3.6 BMSCs‐Exos Promote STAT6 Acetylation and Phosphorylation by Downregulating TRIM24 Expression

**FIGURE 4:**
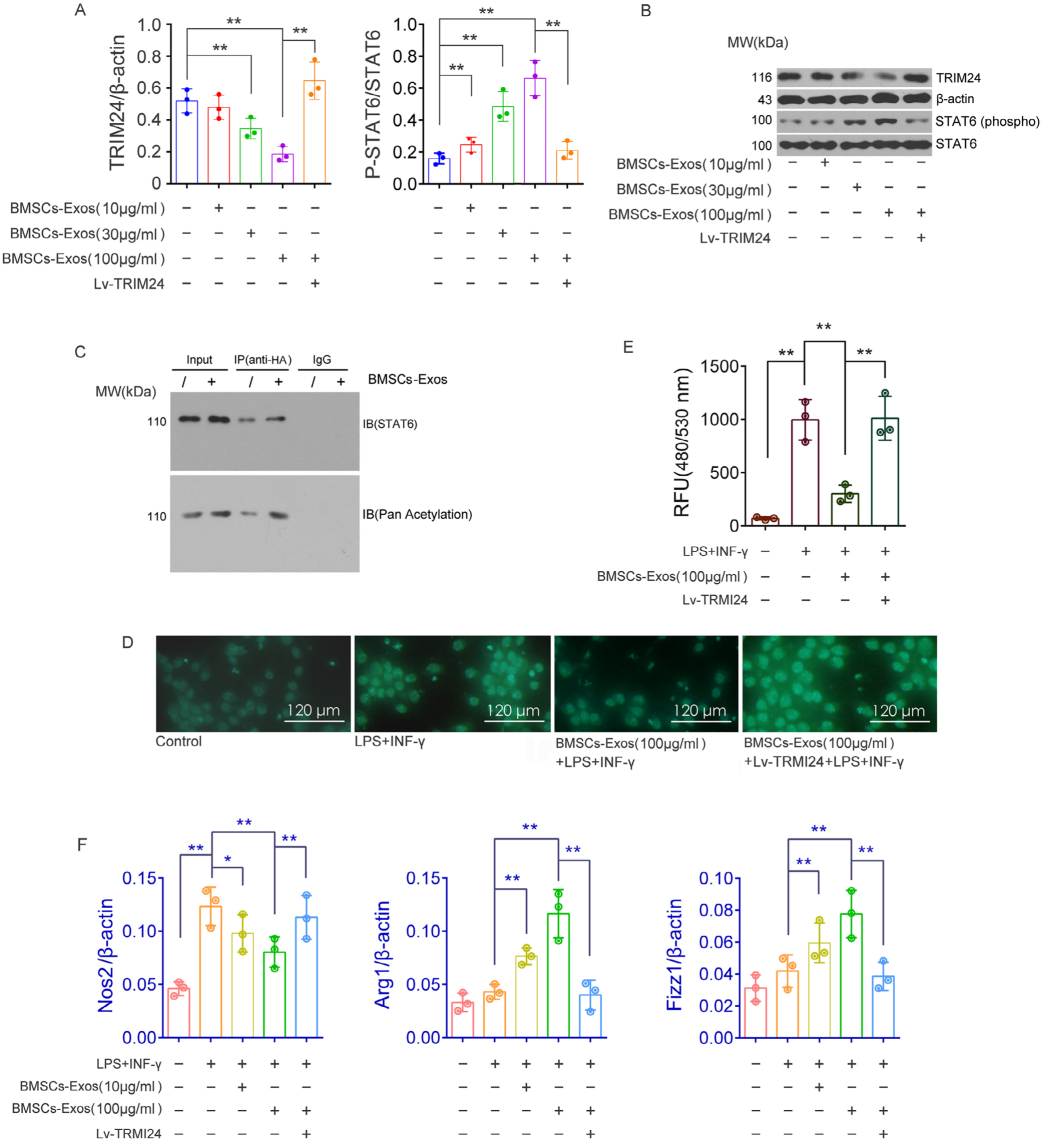
Evaluation of the effects of BMSCs‐Exos on TRIM24 expression, STAT6 acetylation, ROS production, and mRNA levels of macrophage polarization‐related genes in MH‐S cells under different treatment conditions. (A, B) Western blotting was used to assess TRIM24 expression and STAT6 protein phosphorylation in MH‐S cells across treatment groups, with β‐actin and STAT6 as internal controls. (C) STAT6 acetylation changes in BMSCs‐Exos‐treated or untreated MH‐S cells were analyzed by immunoprecipitation. (D, E) ROS activity in MH‐S cells was evaluated across groups. Panel D shows DCF dye fluorescence via inverted microscopy, while Panel E quantifies fluorescence intensity in cell lysates using a UV spectrophotometer (excitation at 480 nm, emission at 530 nm). (F) RT‐qPCR was used to measure the relative expression of Nos2, Arg1, and Fizz1 mRNA in MH‐S cells, with β‐actin as the reference gene. Experiments were conducted with three biological replicates (*n* = 3), with two technical replicates per sample, and data are presented as mean ± SD. *p < 0.05, **p < 0.01, versus the specified group. *t*‐test.

Western blot analysis demonstrated that TRIM24 protein expression in MH‐S cells was significantly downregulated in a dose‐dependent manner following treatment with BMSCs‐Exos at concentrations of 10, 30, and 100 μg/mL, compared to the control group (*p* < 0.01, 30, and 100 μg/mL BMSCs‐Exos vs. control group). Notably, the medium and high dose groups exhibited statistically significant reductions (*p* < 0.01). In contrast, TRIM24 protein expression was markedly increased in MH‐S cells treated with Lv‐TRIM24 + BMSCs‐Exos (100 μg/mL) compared to the BMSCs‐Exos (100 μg/mL) group (*p* < 0.01). In addition, western blotting results also revealed that changes in STAT6 protein phosphorylation within the nucleus showed an opposite trend compared to the alterations in cellular TRIM24 expression across all groups (Figure 4A,B). Immunoprecipitation assays further confirmed that STAT6 acetylation was significantly enhanced after 48 h of treatment with 100 μg/mL BMSCs‐Exos relative to the control group (Figure 4C). These findings are consistent with previous studies reporting that TRIM24 can interact with the acetyltransferase CBP, subsequently suppressing CBP‐mediated STAT6 acetylation. This suppression limits STAT6 transcriptional activity, whereas reduced TRIM24 expression allows for enhanced STAT6 acetylation, thereby promoting STAT6 activation and facilitating M2 macrophage polarization.

### 3.7 BMSCs‐Exos Inhibit ROS Production and Release in MH‐S Cells by Suppressing TRIM24 Expression

The ROS assay revealed that 48 h stimulation with LPS and IFN‐γ significantly elevated ROS activity in MH‐S cells (*p* < 0.01 vs. the control group). However, pretreatment with BMSCs‐Exos (100 μg/mL) markedly suppressed this LPS and IFN‐γ‐induced increase in ROS levels (*p* < 0.01 vs. the LPS + IFN‐γ group). Importantly, the overexpression of exogenous TRIM24 effectively reversed the inhibitory effect of BMSCs‐Exos on ROS activity in MH‐S cells subjected to LPS and IFN‐γ stimulation (*p* < 0.01 vs. the BMSCs‐Exos (100 μg/mL) + LPS + IFN‐γ group) (Figure 4D,E). These findings suggest that BMSCs‐Exos inhibit LPS + IFN‐γ‐induced M1 polarization of MH‐S cells, and this effect is closely associated with the downregulation of TRIM24 expression. This observation is fully consistent with the rationale behind our rescue experiment design, as the exogenous TRIM24 mRNA used lacks the 3′UTR, rendering it insensitive to post‐transcriptional regulation by intracellular miR‐137‐3p.

### 3.8 BMSCs‐Exos Promote M2 Polarization of M1‐Type MH‐S Cells via Activation of STAT6 Transcription Factor

RT‐qPCR analysis revealed that stimulation with LPS+ IFN‐γ for 48 h significantly upregulated the mRNA expression of the M1 polarization marker Nos2 in MH‐S cells (*p* < 0.01 vs. the control group). Compared with the LPS + IFN‐γ group, the mRNA level of Nos2 was decreased in the BMSCs‐Exos (10 μg/mL) + LPS + IFN‐γ group (*p* < 0.05) and further reduced in the BMSCs‐Exos (100 μg/mL) + LPS + IFN‐γ group (*p* < 0.01). Although the mRNA expression of the M2 polarization markers Arg1 and Fizz1 (downstream genes regulated by the transcription factor STAT6) showed an increasing trend following LPS + IFN‐γ stimulation, the differences were not statistically significant (*p* > 0.05 vs. the control group). In contrast, treatment with BMSCs‐Exos (10 and 100 μg/mL) markedly enhanced the mRNA expression of Arg1 and Fizz1 in LPS + IFN‐γ–induced MH‐S cells in a dose‐dependent manner (*p* < 0.01 vs. the LPS + IFN‐γ group). Notably, in the Lv‐TRIM24+ BMSCs‐Exos (100 μg/mL) + LPS+ IFN‐γ group, the mRNA expression of Nos2 was significantly increased, whereas that of Arg1 and Fizz1 was markedly decreased compared with the BMSCs‐Exos (100 μg/mL) + LPS + IFN‐γ group (*p* < 0.01) (Figure 4F). These findings suggest that BMSCs‐Exos promote the repolarization of M1‐type MH‐S cells toward an M2 phenotype, likely through STAT6 activation, and that this effect is negatively regulated by TRIM24.

### 3.9 BMSCs‐Exos Attenuate Pulmonary Injury in ALI Mice

**FIGURE 5:**
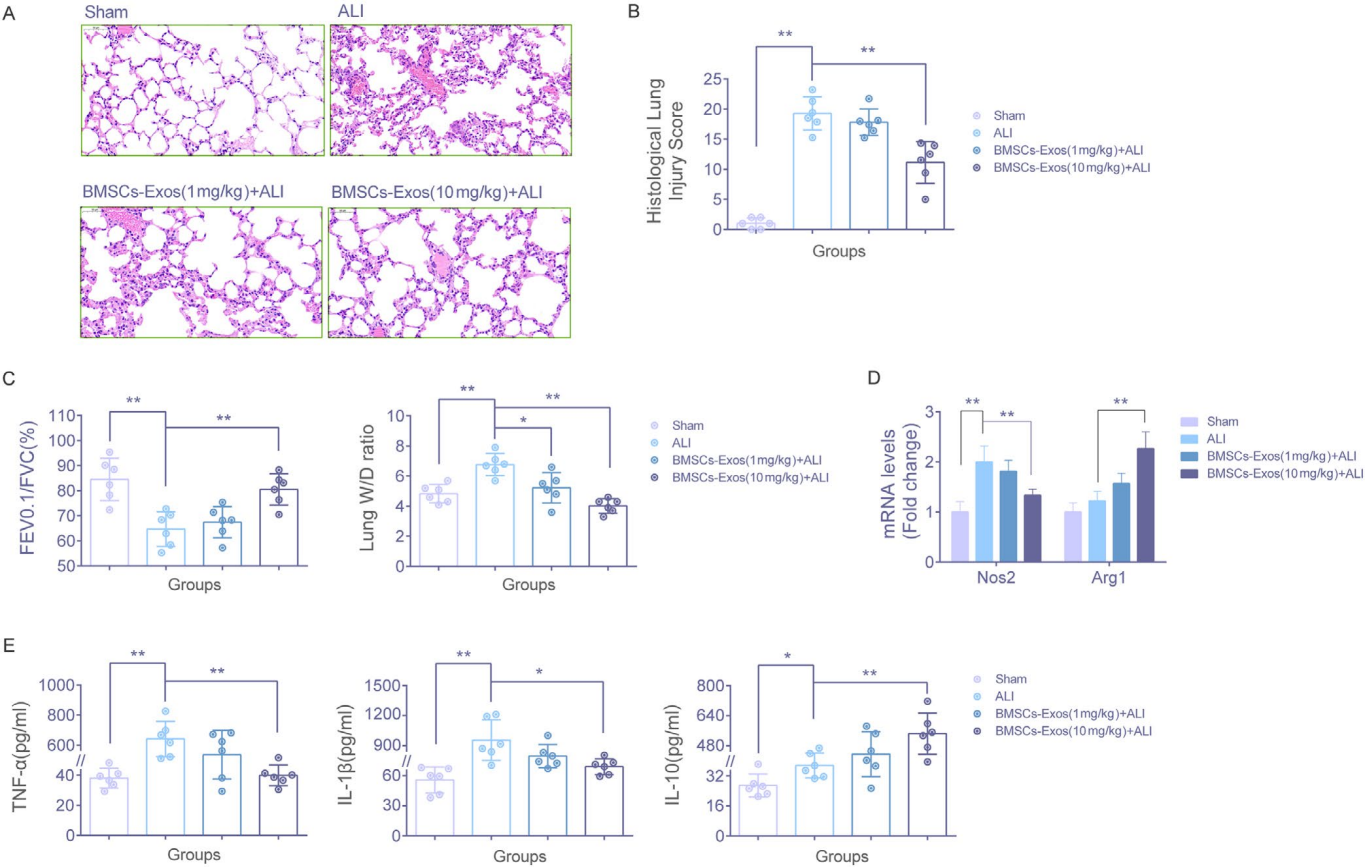
Assessment of lung tissue damage and pulmonary function in ALI mice. (A) HE staining of lung tissue from mice in each group. Images are shown at 200× magnification. (B) Lung tissue damage scores for mice in each group. (C) Evaluation of pulmonary function (FEV0.1/FVC%) and analysis of the wet‐to‐dry weight ratio of lung tissue in each group of mice. (D) RT‐qPCR was used to measure the Nos2 and Arg1 mRNA levels in macrophages derived from the lung tissue of mice in each group. (E) Measurement of inflammatory cytokine (TNF‐α, IL‐1β and IL‐10) levels in the BALF of each group of mice using ELISA. The number of animals per group was set to 6 (*n* = 6), with two technical replicates per sample, and data are presented as mean ± SD. *p < 0.05, **p < 0.01, versus the specified group. *t*‐test.

Histological analysis using H&E staining revealed that, compared with the sham group, lung tissues from ALI mice exhibited pronounced pathological changes, including disruption of alveolar architecture, infiltration of inflammatory cells, hyaline membrane formation, accumulation of exudates, and significant pulmonary edema. Treatment with BMSCs‐Exos at a dose of 1 mg/kg partially alleviated these pathological features, while a higher dose of 10 mg/kg produced more substantial improvements (Figure 5A). Lung injury scores were significantly elevated in the ALI group compared to the sham group (*p* < 0.01). Although a slight reduction was observed in the BMSCs‐Exos (1 mg/kg) + ALI group, the difference was not statistically significant (*p* > 0.05). In contrast, the BMSCs‐Exos (10 mg/kg) + ALI group demonstrated a marked reduction in pathological scores (*p* < 0.01 vs. the ALI group) (Figure 5B). Pulmonary function analysis showed a significant decline in the FEV0.1/FVC (%) ratio in the ALI group compared with the sham group (*p* < 0.01), indicating impaired lung function. This impairment was slightly improved in the BMSCs‐Exos (1 mg/kg) + ALI group without reaching statistical significance (*p* > 0.05). However, the BMSCs‐Exos (10 mg/kg) + ALI group exhibited a significant increase in FEV0.1/FVC (%), suggesting improved pulmonary function (*p* < 0.01 vs. the ALI group). The lung wet‐to‐dry weight ratio, an indicator of pulmonary edema, was significantly increased in ALI mice relative to the sham group (*p* < 0.01). Treatment with BMSCs‐Exos at 1 mg/kg moderately reduced this ratio (*p* < 0.05), whereas the 10 mg/kg dose resulted in a more pronounced and statistically significant decrease (*p* < 0.01 vs. the ALI group) (Figure 5C). RT–qPCR analysis of alveolar macrophages showed that, relative to the sham group, Nos2 mRNA was significantly upregulated in the ALI group (*p* < 0.01), whereas Arg1 exhibited a modest, non‐significant increase (*p* > 0.05). Compared with the ALI group, treatment with BMSCs‐Exos (1 mg/kg) produced a slight decrease in Nos2 and a slight increase in Arg1, neither of which reached statistical significance (both *p* > 0.05). In contrast, BMSCs‐Exos (10 mg/kg) significantly decreased Nos2 mRNA and increased Arg1 mRNA compared with the ALI group (both *p* < 0.01), indicating a dose‐dependent modulation of these polarization‐associated markers (Figure 5D). ELISA analysis of BALF showed significantly elevated levels of pro‐inflammatory cytokines TNF‐α and IL‐1β, as well as the anti‐inflammatory cytokine IL‐10, in the ALI group compared with the sham group (*p* < 0.01 or *p* < 0.05). Although treatment with BMSCs‐Exos at 1 mg/kg led to a slight decrease in TNF‐α and IL‐1β levels and a slight increase in IL‐10 levels, these changes were not statistically significant (*p* > 0.05). In contrast, the 10 mg/kg BMSCs‐Exos treatment group exhibited significant reductions in TNF‐α and IL‐1β, along with a notable increase in IL‐10 (*p* < 0.01 or *p* < 0.05 vs. the ALI group) (Figure 5E).

**FIGURE 6:**
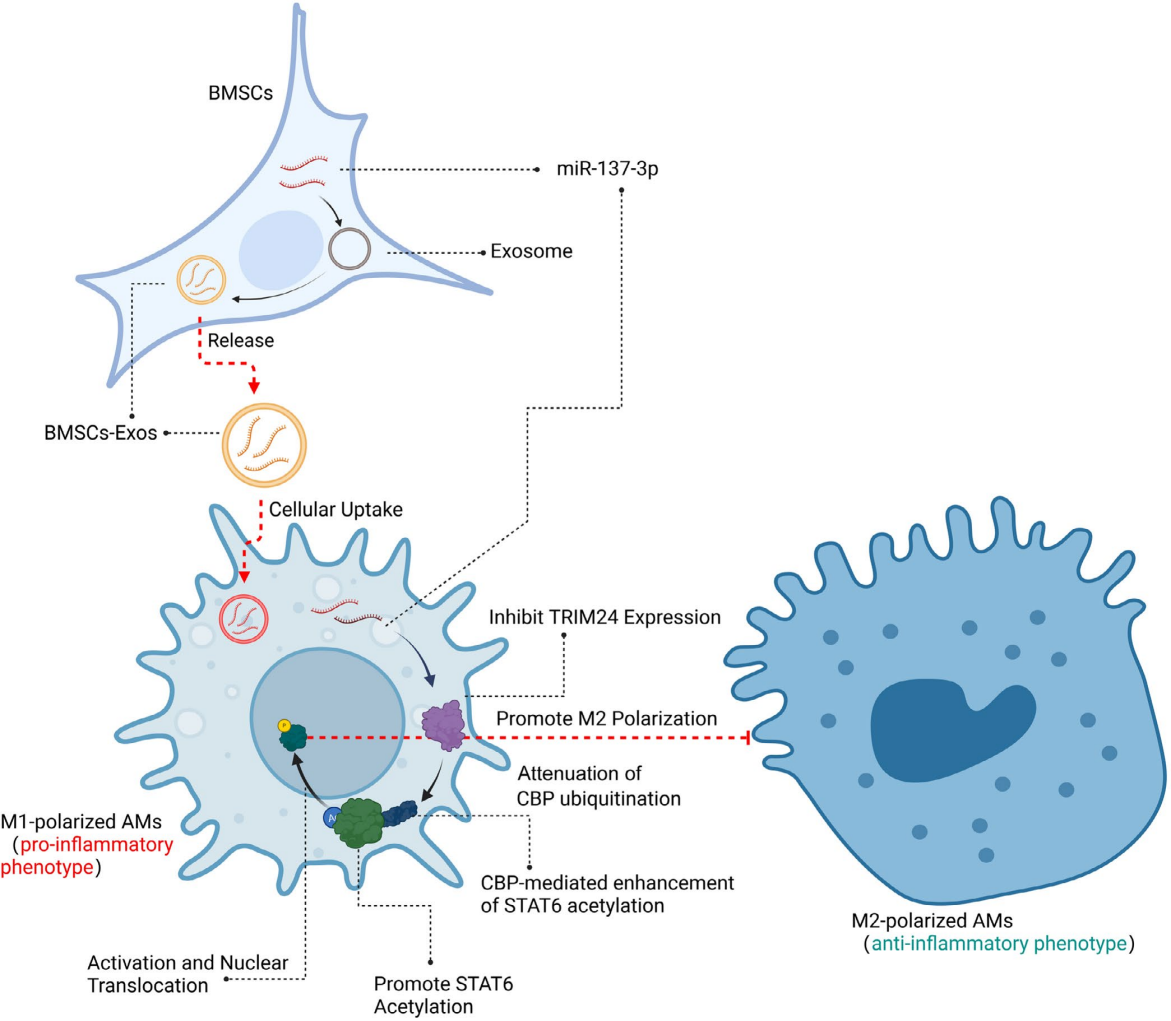
Molecular mechanism. Schematic Representation of the Molecular Mechanism by Which BMSCs‐Exos Regulate M1/M2 Macrophage Polarization to Alleviate Lung Tissue Damage in ALI Mice.

## 4 Discussion

Transplantation of MSCs has shown potential in ALI, sepsis, and acute respiratory distress syndrome (ARDS), with BMSCs improving pulmonary inflammation and lung injury significantly despite the low survival rate of allogeneic transplantation (< 5%) [9]. Previous studies have shown that MSCs could regulate the over‐activated inflammatory response and promote recovery of lung injury by way of immune regulation and regeneration, especially for COVID‐19 patients [19]. However, MSC transplantation faces limitations, including uncertainty in efficacy, variability in clinical outcomes, and factors such as MSC source, administration route, and dosage. MSCs derived from different sources (e.g., bone marrow, umbilical cord, adipose tissue) can lead to inconsistent quality and affect reproducibility. Immune rejection, especially with allogeneic MSCs, remains a concern, and long‐term safety is uncertain, with some studies suggesting a risk of tumor promotion, particularly in immunocompromised patients. Additionally, the survival and distribution of MSCs in local lung tissue are difficult to control, and most transplanted MSCs are eliminated by the recipient's immune system within two days, thus, limiting their therapeutic effects. Given these challenges, extracellular vesicles (EVs) derived from MSCs present a promising alternative. EVs, which carry the therapeutic potential of MSCs, can circumvent some of these issues, offering a safer and more effective approach without the complications of immune rejection or limited MSCs' survival and distribution.

Recent studies suggest that MSC‐derived EVs, particularly exosomes, may offer a more effective and practical alternative for treating lung diseases. Exosomes are enriched with biologically active substances like miRNAs, lncRNAs, and low molecular weight peptides (LMPs), which can be delivered to target cells through endocytosis or receptor‐mediated internalization, modulating cellular pathways and offering a promising therapeutic strategy for lung injuries [20]. Studies have shown that nebulized inhalation of adipose‐derived MSC exosomes in severe COVID‐19 patients improved lung injury, while lung spheroid cell exosomes inhibited pulmonary fibrosis [21, 22]. Exosomes were cell‐derived nanovesicles that participated in the intercellular transportation of important substances, such as small molecule proteins or nucleic acid drugs, which can be delivered to specific cells or tissues, thereby increasing the local concentration of therapeutic drugs and reducing side effects [23]. While the mechanisms of exosome therapy still need more research, their effectiveness in anti‐inflammatory, anti‐fibrotic, and tissue repair, as well as low toxicity, low immunogenicity, and high engineerability processes highlights their potential as a promising and clinically viable treatment for ALI.

Despite these promising developments, the role of MSC‐derived exosomes in macrophage polarization via microRNA delivery has not been fully explored in the development process of ALI. In this study, we identified miR‐137‐3p from exosomes derived from mouse BMSCs‐Exos as a key player in regulating alveolar macrophage polarization. We initially observed that co‐culturing BMSC‐derived exosomes with alveolar macrophages led to macrophage reprogramming and a significant downregulation of TRIM24 expression. While TRIM24 protein levels decreased, mRNA levels remained unchanged, suggesting post‐transcriptional regulation. Given that miRNA‐mediated suppression is a known mechanism, we hypothesized that a specific miRNA might target TRIM24 mRNA. Instead of an unbiased miRNA screen, we used bioinformatics tools to identify miRNAs with binding sites in the 3′UTR of TRIM24. The predicted miRNAs were validated through correlation analysis, dual‐luciferase assays, and other techniques, ultimately identifying miR‐137‐3p as the key regulatory miRNA. As expected, we subsequently detected the presence of miR‐137‐3p in BMSC‐derived exosomes.

Mouse miR‐137, located on chromosome 1 (Chr1: 184 720 141–184 720 227), is highly conserved across mammals, with significant sequence similarity between mice and humans. MiR‐137‐3p is widely involved in various biological processes, including neurodevelopment, immune regulation, and cancer suppression. To date, no specific research has been conducted on its role in ALI pathology or in regulating alveolar macrophage polarization. Given its tumor‐suppressive effects in neurodevelopment and cancer, the potential application of miR‐137‐3p in the treatment of ALI warrants further exploration. Our results show that miR‐137‐3p, derived from BMSCs, can be delivered to co‐cultured mouse alveolar macrophages via exosomes. In doing so, it upregulated M2 polarization factors by inhibiting its target protein TRIM24, acetylates STAT6 protein, and promotes the polarization of alveolar macrophages from M1 to M2 while suppressing M1 polarization. Further animal experiments demonstrated that BMSC‐exos, through miR‐137‐3p delivery, mitigated inflammatory lung tissue damage and improved lung function in ALI mice effectively. These findings highlight the therapeutic potential of MSC‐derived exosomes, particularly those containing miR‐137‐3p, in regulating macrophage polarization and alleviating ALI.

M1 macrophages promote inflammation, while M2 macrophages, induced by Th2 cytokines, are associated with anti‐inflammatory responses. Studies showed that BMSCs‐Exos enhanced skin wound healing by promoting M2 polarization via miR‐221‐3p delivery, and UC‐MSC‐derived exosomes improved wound healing in diabetic patients by inducing M2 polarization [24]. Recent research also suggested exosomes derived from hUC‐MSC exerted anti‐inflammatory and immunomodulatory effects and inhibited macrophages proliferation by restraining M1 macrophages and inducing M2 macrophages polarization [25]. However, understanding the specific active substances in MSC‐derived exosomes is crucial for their therapeutic applications, especially for lung injury treatment. In the long term, to expand the clinical use of MSC‐derived exosomes in ALI indicators, an engineered strategy to prepare functional EVs containing active substances, instead of extracting from mesenchymal cells, is a necessary alternative. In this study, we identified miR‐137‐3p in mouse BMSCs‐Exos as a key regulator of alveolar macrophage polarization. BMSCs‐Exos deliver miR‐137‐3p to alveolar macrophages, suppress TRIM24 expression, enhance STAT6 acetylation, and promote M2 over M1 polarization. Although there is no direct evidence confirming that TRIM24 regulates STAT6 acetylation currently, based on the molecular functions of TRIM24 and the regulatory mechanisms of STAT6, it can be reasonably speculated that TRIM24 may negatively regulate STAT6 acetylation and its transcriptional activity [26]. This effect may be achieved through competitive inhibition of acetyltransferases (such as CBP/p300), recruitment of deacetylases (such as HDAC3), or mediation of ubiquitination and subsequent degradation of STAT6. As a result, TRIM24 could suppress macrophage M2 polarization (e.g., reducing the expression of Arg1/Ym1). This study suggests that BMSCs‐Exos regulate the TRIM24/CBP/STAT6 axis via miR‐137‐3p delivery and targeting the TRIM24‐STAT6 acetylation pathway may provide a promising therapeutic strategy for ALI.

In our study, we found that the addition of mouse BMSCs‐Exos effectively facilitates STAT6 acetylation and promotes M2 polarization in MH‐S cells. This effect of BMSCs‐Exos is dependent on miR‐137‐3p delivery and the subsequent suppression of TRIM24 expression at the post‐transcriptional level. While many studies have reported that miRNAs can directly target TRIM24 and regulate its roles in cell proliferation, tumor formation, and inflammation [27, 28], this is the first identification of the macrophage polarization regulation pathways miR‐137‐3p/TRIM24/STAT6. This discovery provides new insights into the complex regulatory network between miRNAs and TRIM24 and offers potential therapeutic targets for diseases such as ALI.

This study highlights the potential of BMSCs‐Exos in promoting M2 polarization and inhibiting M1 polarization of alveolar macrophages through miR‐137‐3p‐mediated regulation of the TRIM24/CBP/STAT6 pathway, offering an effective approach for alleviating ALI. This discovery provides new insights into the precision immunomodulation of alveolar macrophages, with significant clinical potential for exosome‐based therapies. Exosomes, as key mediators of intercellular communication, can deliver therapeutic miRNAs directly to target cells, overcoming challenges associated with traditional MSC transplantation, such as immune rejection and poor cell survival. Compared to conventional cell therapy, exosome‐based treatment offers enhanced safety and controllability, particularly in the treatment of lung injury.

Looking ahead, exosome‐based therapies hold promising prospects in the treatment of lung injuries. By precisely regulating alveolar macrophage polarization, exosomes can exert anti‐inflammatory, anti‐fibrotic, and tissue repair effects, making them a potentially effective and scalable therapeutic option. Furthermore, with advances in exosome engineering, the targeted delivery and modification of their contents can enhance their therapeutic efficacy, enabling precision‐targeted treatments for a wide range of acute inflammatory diseases. This approach holds substantial clinical promise for expanding the treatment options for ALI and other inflammation‐driven conditions.

In our ongoing research, we recognize the critical need to enhance the therapeutic efficacy of BMSCs‐Exos for treating diseases driven by acute inflammatory injury, such as ALI, and beyond. We are focusing on two key strategies currently. First, our team is working to increase miR‐137‐3p expression in BMSCs through gene insertion or targeted induction, or by loading exosomes with miR‐137‐3p using in vitro chemical synthesis, thereby enriching the miR‐137‐3p content in BMSCs‐Exos. Additionally, we are employing click chemistry to integrate receptors onto the phospholipid membrane of BMSCs‐Exos, enabling specific recognition of macrophage surface proteins, which is essential for precision‐targeted biological therapies. We believe these efforts will significantly enhance the clinical efficacy of BMSCs‐Exos in treating a variety of acute inflammatory injury‐driven diseases, extending beyond just ALI. Furthermore, our findings provide a solid foundation for developing standardized biological therapies using BMSCs‐Exos to address a wide range of inflammatory conditions. In summary, this study demonstrates that BMSCs‐Exos promote M2 polarization and suppress M1 polarization of alveolar macrophages via miR‐137‐3p mediated regulation of the TRIM24/CBP/STAT6 pathway (Figure 6 illustrates the mechanisms) suggesting their potential as a novel therapeutic strategy for ALI.

## Author Contributions

Yingchuan Li, Yongmei Cao and Kaixuan Feng conceptualized the study. Yongmei Cao and Weifeng Huang developed the model, while Yongmei Cao, Weifeng Huang, Jiawei Shang, Feng Ping, Qin Tan, and Wei Wang carried out the experiments. Weifeng Huang and Yingchuan Li conducted the data analysis. Kaixuan Feng drafted the manuscript, and Yongmei Cao and Yingchuan Li revised it. All authors have reviewed and approved the final version of the manuscript for submission.

## Funding

This work was financially supported by the National Natural Science Foundation of China (82272245 and 82402563); Shanghai Chongming District Sustainable Development and Technological Innovation Action Plan Project (CKY2024‐36).

## Consent

The authors have nothing to report.

## Conflicts of Interest

The authors declare no conflicts of interest.

## Data Availability

Data will be made available on request.
